# Identifying applications of virtual reality to benefit the stroke translational pipeline

**DOI:** 10.1177/23982128231182506

**Published:** 2023-06-21

**Authors:** Matan Bone, Maham Malik, Siobhan Crilly

**Affiliations:** 1 School of Medical Sciences, Faculty of Biology, Medicine and Health, Manchester Academic Health Science Centre and The University of Manchester, Manchester, UK; 2 Division of Neuroscience, School of Biological Sciences, Faculty of Biology, Medicine and Health, Manchester Academic Health Science Centre and The University of Manchester, Manchester, UK; 3 Geoffrey Jefferson Brain Research Centre, Manchester Academic Health Science Centre, Northern Care Alliance and The University of Manchester, Manchester, UK

**Keywords:** Translational research, stroke, animal model, computer simulation, clinical research

## Abstract

As a leading cause of mortality and morbidity, stroke and its management have been studied extensively. Despite numerous pre-clinical studies identifying therapeutic targets, development of effective, specific pharmacotherapeutics remain limited. One significant limitation is a break in the translational pipeline – promising pre-clinical results have not always proven replicable in the clinic. Recent developments in virtual reality technology might help generate a better understanding of injury and recovery across the whole research pipeline in search of optimal stroke management. Here, we review the technologies that can be applied both clinically and pre-clinically to stroke research. We discuss how virtual reality technology is used to quantify clinical outcomes in other neurological conditions that have potential to be applied in stroke research. We also review current uses in stroke rehabilitation and suggest how immersive programmes would better facilitate the quantification of stroke injury severity and patient recovery comparable to pre-clinical study design. By generating continuous, standardised and quantifiable data from injury onset to rehabilitation, we propose that by paralleling pre-clinical outcomes, we can apply a better reverse-translational strategy and apply this understanding to animal studies. We hypothesise this combination of translational research strategies may improve the reliability of pre-clinical research outcomes and culminate in real-life translation of stroke management regimens and medications.

## Introduction

Stroke is a pathological condition whereby brain damage occurs due to insufficient blood supply caused by either a blockage in the cerebrovasculature or a rupture and haemorrhage into the brain tissue. In 2019, stroke was the second leading cause of disability-adjusted life-years (DALYs) for both the 50–74 and 75+ years of age groups (GBD 2019 Diseases and Injuries Collaborators, 2020). Common sequelae include neurological and physical deficits, such as hemiparesis, cognitive deficits, hemianopsia, the inability to walk unassisted and bladder incontinence (Kelly-Hayes et al., 2003). In the United Kingdom, patients facing disability after a stroke are offered at least 45 min of restorative therapy at least 5 days per week (National Institute for Health and Care Excellence (NICE), 2013). Restorative therapies are determined by the key individual deficits faced by the patient post-stroke, and can include physical therapy, occupational therapy, and speech and language therapy. The global and individual burden of stroke and rehabilitation for patients highlights the desperate need for effective therapies that prevent brain damage and thus protect against long-term disability.

Currently, the first-line treatment for acute ischaemic stroke patients involves the use of recombinant tissue plasminogen activators (rtPA), such as alteplase (NICE, 2019), a protocol first developed in the 1980s and not substantially altered since. However, despite the therapeutic efficacy rtPA therapy presents clinical problems, such as a narrow therapeutic window of 4.5 h, risk of early death and haemorrhagic transformation (Brown et al., 2013) and restrictions to those who can be treated as it cannot be used in patients with intracerebral haemorrhage (ICH) and so diagnosis must be definite (NICE, 2019). Recently, there has been an increase in evidence suggesting the benefits of alteplase beyond the therapeutic window in cases where there is a Diffusion-Weighted Imaging Fluid-Attenuated Inversion Recovery (DWI-FLAIR) mismatch (Berge et al., 2021; Thomalla et al., 2020). Currently, there are no specific medical therapies for haemorrhagic stroke patients, and clinical treatment revolves around surgical intervention and alleviation of underlying aetiology, such as hypertension and anticoagulation therapy (Parry-Jones et al., 2019) and symptom management in an effort to decrease intracranial pressure (NICE, 2019).

Despite the discovery of many potential therapeutic targets, such as N-methyl-D-aspartate (NMDA) receptor antagonists (Muir and Lees, 1995), interleukin-1 receptor antagonist and the post-stroke inflammatory response (Salmeron et al., 2019; Smith et al., 2018) and antiplatelet therapy (Sandercock et al., 2008), advances in stroke therapy over the past 30 years have been minimal, particularly due to a break in the translational pipeline: the promising results of new drugs in pre-clinical studies do not translate into clinical success (Withers et al., 2020). An analysis of thrombolytics and neuroprotective drugs found that only 4% of the agents undergoing trials between 1995 and 2015 reached the market (Chen and Wang, 2016). This translational failure means that stroke has remained within the top four causes of death in the United Kingdom for the past two decades (World Health Organisation, 2020).

**Figure 1.**
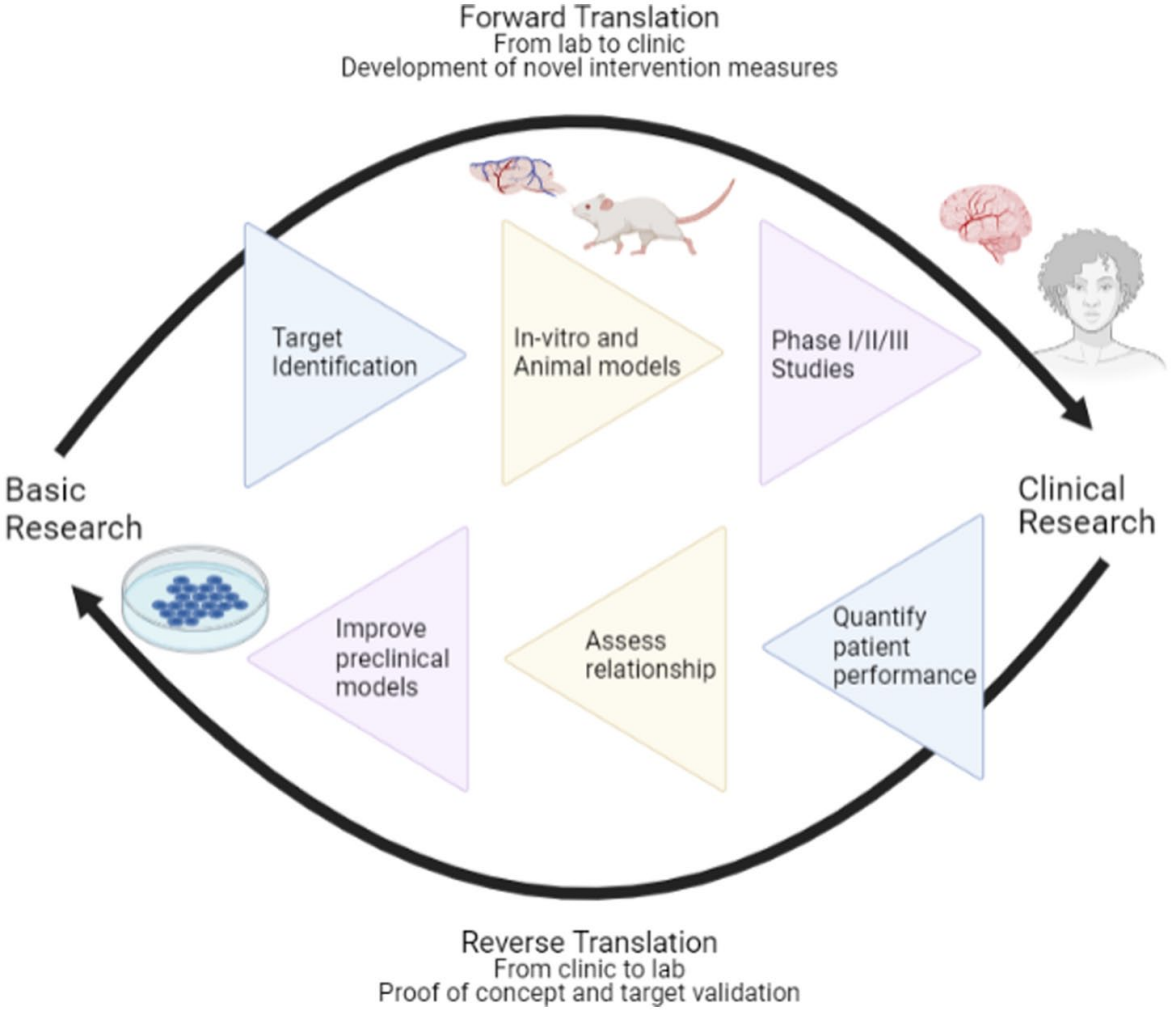
Reverse translation as a means of advancing pre-clinical and clinical stroke research. Source: Created with BioRender.com.

Improving the translatability of drugs requires an improvement to the drug discovery pipeline both in pre-clinical studies and interpretation of clinical outcomes. Many attempts to refine the pre-clinical pipeline have already been made and reviewed extensively (Fisher et al., 2009; Kumar et al., 2016; Lourbopoulos et al., 2021; MacRae, 2011). Here, we review efforts in reverse translation: applying clinical data to influence animal studies, potentially with the use of virtual reality (VR) to standardise data from patients (Figure 1) (Lee et al., 2021).

VR technology facilitates the simulation of an alternative environment either through immersive or non-immersive means, allowing the user to perceive and interact with a reality that may include features or objects absent from their physical environment. Clinical application of this technology has previously shown success in the treatment and quantification of parameters in neurological diseases. Here, we look at the possibility of using VR for the quantification of post-stroke assessment of human patients to measure stroke severity and the therapeutic efficacy of drugs. We hypothesise that these data can be used in a reverse translation approach to refine pre-clinical studies and improve the effort to develop novel, clinically relevant stroke therapeutics.

## Methodology

The authors searched for current literature based on a set of search terms: ‘translational medicine’, ‘reverse-translational approaches’, ‘virtual reality’, ‘stroke modelling’ and ‘stroke rehabilitation’ in the context of neuroscience research. Searches were conducted up until October 2022 on PubMed MEDLINE. Case reports and opinion pieces were excluded. When referring to clinical guidelines, the authors utilised the National Institute of Clinical Excellence (NICE) guidelines (NICE, 2013, 2019).

## A translational problem

Failures in the translational pipeline have been reviewed extensively elsewhere, however for stroke in particular, current clinical practice does not parallel with the pre-clinical strategy of measuring stroke severity and outcome. Changes to practice in both the laboratory and the clinic can be made to increase the likelihood that data collected from both models and patients are better comparable. To measure stroke severity pre-clinically, both ischaemic and haemorrhagic stroke models tend to measure cerebral histological changes such as infarct volume and peri-haematomal area respectively, and conduct motor tests to measure spatial awareness, memory and fine motor skills. However, motor tests are not always consistently carried out between studies, and some do not have parallels between mice and rats. Contrastingly, discrete classification in clinical testing such as the Glasgow Coma Scale (GCS), National Institute of Health Stroke Scale/Score (NIHSS) and modified Rankin Scale (mRS) is less sensitive to changes during stroke recovery. In addition, such tests are mainly behaviour-focussed assessments that measure multiple aspects of a post-stroke deficit such as language, gross and fine motor response, and ability to carry out Activities of Daily Living (ADLs), and so are unable to be measured equivalently in rodent models. The difference between pre-clinical assays and clinical tests means that comparing outputs across species is complex and not always indicative of a drug’s therapeutic power, contributing to the stall in drug discovery.

The need for novel therapies for stroke is compounded by the increasing incidence of risk factors in the general population, such as ageing and hypertension (Mills et al., 2020; Woo et al., 2004). However, the development of novel drugs through the translational pipeline continues to fail. Examples include recombinant erythropoietin (EPO) for ischaemic stroke, where EPO and EPO analogues were associated with a decrease in infarct size and an improvement in various behavioural tests using multiple stroke models (Minnerup et al., 2009). In clinical trials however, the EPO treatment arm showed an increase in complications, including ICH and cerebral oedema and caused a two-fold increase in early mortality of patients (Ehrenreich et al., 2009). Consequently, although significant reductions in lesion sizes were observed in the dual treatment rtPA with EPO cohort, this did not translate into significant improvements in clinical tests including the Barthel Index (BI), mRS and NIHSS all conducted at days 30 and 90 post-enrolment. This is a similar case for BAYx3702 (Repinotan), a serotonin 1A receptor (5-HT1A) agonist, which was observed as causing the reduction of infarct size in multiple murine models (Berends et al., 2005). However, a clinical trial of BAYx3702 showed no statistically significant difference between the NIHSS, BI and mRS of the placebo and treatment group (Teal et al., 2009). In addition, deferoxamine, an iron chelator trialled for post-ICH therapy showed very promising results in pre-clinical models in a comparable manner (Selim, 2009), but high doses were associated with adverse reactions in clinical trials (hi-DEF), and progression was deemed futile (Selim et al., 2019).

In the following sections, we will discuss how implementing VR into quantifying patient outcomes to reverse translate to pre-clinical studies may help bridge the translational gap, with the potential to increase the proportion of successful stroke therapies entering development.

## Current translational approaches

Common rodent models of ischaemic stroke and haemorrhagic stroke reflect the human condition to varying extents and tend not to mimic clinical populations where patients exhibit comorbidities (Bhagavati, 2015) including hypertension, diabetes, cerebral amyloid angiopathy and advanced age (Turner et al., 2016). These are complex conditions to model and can confound the consistent outcome of surgical stroke. Therefore, limiting pre-clinical parameters to measure infarct size as an indication of drug efficacy in ischaemic stroke (Turner et al., 2016) or size/location of haematoma and cerebral oedema (MacLellan et al., 2012), is not always clinically relevant. There are wider deficits in patients due to white matter damage that cannot be replicated in rodents, and therefore, reverse-translational approaches are necessary to better the pre-clinical models for stroke

Clinically, assessments of post-stroke neurological abilities focus on physical or cognitive deficit. Some of the more commonly used clinical tests, such as the GCS, NIHSS and the mRS, output a single number based on the assessment of both physical and cognitive performance post-stroke. These assessments have low sensitivity and rely on patient interpretation of their own performance and abilities. When compared to pre-clinical assays, there are few direct parallels. One example of such a translatable test is the Tübingen–Boston Rat Coma Scale (RCS) developed by Pais-Roldán et al. (2019) who used an endothelin-1 injection to cause brain infarctions and induce coma, after which the animals were analysed for behavioural outcomes in the acute stages of coma recovery. The RCS is an example of a GCS-like stroke scale which quantifies the following parameters: eye blinks, motor function, brainstem reflexes, respiration, righting reflex, auditory response and whisker movement. Animals do not need to be trained prior to injury (as is required for behavioural assays), and so the outcomes are more similar to human assessment. Interrogating a range of responses may provide increased sensitivity to damage in different brain regions, rather than interrogating individual gross motor skills in commonly used behavioural assays. On the contrary, determining outcomes using the RCS is open to inter-researcher variation which may limit standardisation; however, this mirrors the heterogeneous recovery of patients’ post-stroke and the similar subjectivity of the GCS. Although thus far not employed in stroke research, we hypothesise that this scale or a derivative could be standardised in rat-model stroke studies to monitor the post-stroke severity with and without therapeutics. Alternatively, instead of adapting pre-clinical investigation to resemble clinical outputs, we can develop strategies to assess patients in ways similar to animal behavioural assays.

## Potential for VR in reverse translation

We hypothesise that by increasing the sensitivity in clinical assessments, and by interrogating outcomes similar to those in pre-clinical assays, we can generate more comparable data across the pipeline. One such strategy to develop more translatable measures after stroke may be to employ VR technology to quantify and standardise measurements of patient performance (Figure 1) to draw parallels with the quantitative data that are acquired from pre-clinical assays. VR technology facilitates the simulation of an alternative environment either through immersive or non-immersive means, allowing the user to perceive and interact with a reality that may include features or objects absent from their physical environment. In the literature, VR content can be delivered both via 2D screens and head-mounted displays and can include auditory stimulus for a more immersive experience. Systems that include tracking technology, such as hand-held controllers or specialist equipment vary largely and therefore might be less possible to scale up. Although the audio/visual VR scenarios cannot be applied to rodents, the technology and data algorithms could potentially be used to develop robust, reproducible quantitative data from patients, which we hypothesise to be comparable to known rodent behavioural assays. VR and tracking technology would increase sensitivity in detecting changes in patient performance, in addition to discrete classification data. This would add greater value to clinical measurements, allowing for the collection of more data that are easier to correlate with their pre-clinical counterparts. In addition, some systems can be used to interrogate other cognitive functions after injury, such as memory and anxiety-related disorders. Cipresso et al. (2018) found that the application of VR in neuroscience and neurology has increased from 12% to 18.6% from 2011 to 2016, highlighting the increased involvement of this tool in clinical practice, and the potential to expand its range of uses in healthcare.

## Impact of treatments and rehab strategies for stroke patients

Specifically in stroke research, thus far the use of VR has been predominantly rehabilitative rather than assessment-focussed, with a particular focus on upper limb rehabilitation. A Cochrane review analysing the use of VR in the post-stroke rehabilitation of the upper limb found that it was not a more effective treatment option compared to standard care, and that the studies included were classed as low/moderate quality of evidence based on the GRADE system (Laver et al., 2017). Although there was an improvement when used as an adjunct to conventional therapy, this may be due to the overall increase in time spent in therapy compared to control groups rather than proof of the efficacy of VR in upper limb rehabilitation.

The BTS–Nirvana VR device is an example of a minimally intrusive sensor used for rehabilitative purposes which utilises optoelectronic infrared sensors (De Luca et al., 2018) to monitor user movements in response to audio-visual, locomotor and cognition prompts from interactive games, after which the system provides feedback to measure attention, verbal memory and visuospatial abilities. This technology was developed specifically to aid in the rehabilitation of motor function in those with neuromotor disorders, including stroke, Parkinson’s disease (PD) and multiple sclerosis (BTS Bioengineering, 2019). A specialist system like this might be difficult to scale up and employ in multiple stroke centres, however alternatively, having only one group that can employ the BTS–Nirvana device might promote centralisation of the data and better access for pre-clinical researchers. Measures of patient attention could potentially present a model for comparison with rodent novel object recognition (NOR) assessments. The non-invasive infrared sensor technology could also be adapted to rodent movements, albeit with different cognitive stimuli in an effort to improve the quantification of post-stroke deficit in animal trials.

In addition to motor rehabilitation, Faria et al. (2016) demonstrated the efficacy of VR in post-stroke rehabilitation of cognitive deficits in a randomised controlled trial of 18 patients using a 3D virtual simulated city, known as Reh@City, to facilitate imitated ADLs. Each task measured various aspects of cognitive ability, such as memory, attention and visuospatial orientation; and with each task completed around Reh@City the user would accumulate points, with points deducted for mistakes or usage of the ‘help’ button. Overall, the results describe the experimental VR group showing significant improvements in post-intervention in attention, memory and visuospatial abilities compared to the control group.

Regarding the treatment of wider neurological conditions, VR was found to be more effective than standard care in treating depression, anxiety, pain and fatigue (Ioannou et al., 2020). One example of how VR has been used is in delivering cognitive behavioural therapy (CBT) in the treatment of generalised social anxiety disorder via a head-mounted display simulating a busy environment, and allowing patients to engage in CBT with alteration of the virtual environment by the presence of a therapist (Geraets et al., 2019), which could provide a controlled exposure (unlike in-person experiences) to potentially stressful situations which can also be built upon and discussed further with therapists.

Similarly, VR has been used in physical therapy of paediatric patients suffering from burns to reduce subjective pain associated with burn rehabilitation therapy (Schmitt et al., 2011). This VR simulation utilised a previously designed 3D environment with head-mounted displays where the users interact by throwing snowballs at targets, which is a prime example of the potential of customising VR environments according to patient demographic. These successes highlight the potential of VR in the use of other conditions such as stroke, as it demonstrates the effectiveness of VR in both physical and mental conditions which may pave the way to quantifying mental capacities in memory and attention, and physical ability, such as limb strength.

## Application to assessment of injury severity

Based on the methodology and results of the rehabilitation methods described above, it can be proposed that similar mechanisms can be used to quantify memory, unilateral spatial neglect (USN), coordination and motor function to use as analogues to pre-clinical behavioural tests that focus on assessing corresponding deficits occurring after a stroke

A study with simple applications was conducted by Rubio et al. (2013). The study recorded limb kinematics to record four parameters for each upper limb: work area, distance covered, fingers flexion and fingers extension. On analysis, the study found that the distance moved parameter significantly correlated with four clinical tests included in the study: Medical Research Council Scale for proximal upper limb (MRCp), Ashworth Scale for proximal upper limb (ASp), BI, and Fugl–Meyer Motor (FM) assessment. Similarly, there were also correlations between finger movement (flexion/extension) and other scales including FM, the Medical Research Council Scale for distal upper limb (MRCd) and the Ashworth Scale for distal upper limb (ASd). These results propose that a rehabilitation gaming system may be able to accurately assess the motor function of a stroke patient across clinical scales similar to fine motor testing in pre-clinical rodent models, such as skilled reaching.

Similar programmes have been developed for neurological assessment. For example, Choi et al. developed a 2D-screen mobile game-based VR tool to aid with rehabilitation of the upper limb (Choi and Paik, 2018), which consisted of four games targeting a variety of movements: shoulder adduction and abduction, and elbow flexion and extension. The system monitors the movement of the participant’s upper limb using sensors present in a smartphone attached to the participant’s arm which transfers collected data to a tablet PC. Data from these games that quantify range of mobility and strength could be used to scale hemiparesis after stroke and recovery over time in humans, and draw parallels with grip strength and lateral preferences determined by cylinder/corner tests in rodents.

Recently, a case–control pilot study was conducted by Zúñiga et al. (2021) assessing post-stroke motor function of the trunk and upper limb using a 3D avatar of the body to monitor movement without the attachment of markers to the subject, which showed that the VR quantification was more sensitive than clinical scales and found a correlation between the mRS and degree of body sway. Another example of VR application providing useful quantification of functional outcomes is the work of Isenstein et al. (2022), whereby the authors used hand tracking VR to assess both paired visual-proprioceptive ability and isolated proprioception by making the reaching virtual hand invisible. The results of the test showed that there was a statistical significance between accuracy when the hand was visible, compared to invisible, allowing isolated proprioception to be measured sensitively within the test population. This allowed the authors to quantify a previously categorical measure of the finger-to-nose test for upper limb coordination. These data support the use of VR in post-stroke assessment, to quantify severity with a view to producing translational data equivalent to assays, such as the foot fall test and cylinder test.

## Investigating comparisons with pre-clinical assessments

Crucially, adaptations to pre-existing VR programmes must closely follow pre-clinical behavioural tests without losing ecological validity or the ‘game’ aspect that promotes gratification, motivation and engagement. An example of this may include developing a simulated task specifically tailored to spatial memory and locomotor function resembling the Morris Water Maze (MWM), or memory and anxiety, such as the NOR with the programme being set in a real-life situation, such as a simulation of an ADL, similar to Reh@City (Faria et al., 2016). There are currently programmes that allow for the simulation of various virtual mazes using immersive VR, including parallels to the MWM, T-maze and radial arm maze using head-mounted displays and hand-held controllers (MazeEngineers, 2018). This technology has been used successfully to measure spatial memory studies of other neurological disorders, such as PD, where cognitively impaired patients were found to have significantly reduced performance compared to controls in virtual water maze tasks, but similarly to non-cognitively impaired PD patients (Schneider et al., 2017). These programmes could easily be applied to stroke patient clinics to measure injury severity with direct pre-clinical comparison to validate animal models. Similar technology has previously been used to assess navigational impairment in traumatic brain injury (TBI) (Livingstone and Skelton, 2007) using an analogue of the MWM in lieu of a traditional layout. This study demonstrated that the navigation of the TBI group was impaired when proximal markers were absent, similar to a MWM assessment. Quantitative measures for both studies included total distance moved and latency to reach target (Livingstone and Skelton, 2007; Schneider et al., 2017), whereas the PD study measured average accuracy of path towards target as a third output where the TBI study quantified dwell time, each applicable to stroke patients and easily assessed in rodent assays.

A key limitation of using VR clinically relates to the scalability which may prove to be hindered by access to reliable supportive technology including monitors, a reliable source of electricity and the cost of equipment. An estimate of cost for one VR MWM with full equipment is 5290 USD (MazeEngineers, 2018). Similarly, to implement VR-guided stroke assessment, the training of both patient-facing staff and technology-based staff will be required to ensure continuous and safe working of equipment. While these are largely problems that occur at the beginning of scheme implementation, a long-term issue that may be encountered is patient uptake in long-term VR sessions and the engagement of elderly populations who may have an aversion to VR.

Another aspect to consider regarding the reverse-translatability of outputs is the standardisation of sensitivity between different softwares and equipment, as this could alter the assimilated results reaching pre-clinical trials. This is a problem currently present in forward translation where the loss of standardised pre-clinical tests affects the translation of drugs from animal to human subjects. Therefore, in order for reverse translation to objectively target this shortcoming and bridge the translational gap, this must be addressed before the use of VR outputs in stroke research.

The pre-clinical focus remains on finding biological targets rather than assessing the feasibility of targeting them using therapeutics causing a lower clinically relevant output despite increased research funding (Butler, 2008). An example of this disconnect between clinical and pre-clinical research is regarding the time window at which treatment is received between subjects in both study types, which is a well-known indicator of recovery in stroke patients receiving rtPA therapy or thrombectomy. Rodents in studies tend to achieve recanalisation far sooner than is achievable for humans in clinical practice (Lourbopoulos et al., 2021). This may lead to patients falling outside the window where treatment is effective. The ability to better compare these models with clinical outputs gives researchers the opportunity to identify which behavioural tests in animal models would be most likely to signify patient improvement or drug efficacy. This strategy could potentially refine animal tests and reduce the numbers required by generating more rigorous data, a 3Rs aim of pre-clinical research.

## Conclusion

Here, we have discussed the hypothesis that adaptations to the translational pipeline may increase sensitivity in translation between animal and clinical data and ultimately advantage the development of stroke therapeutics that has remained stagnant for so long. VR has shown promise as a method for reverse translation in stroke research, particularly for USN and upper limb function for which VR-based scores have correlated with clinical assessment scores. With modification of programmes already in use and availability of clinical data sets from rehabilitation, VR may allow for the reproducible quantification of multiple post-stroke assessments ranging from motor to cognitive function to parallel the pre-clinical investigation. To validate the comparability of clinical data generated using VR in this manner, quality randomised control trials with large sample sizes using VR to assess multiple post-stroke deficits are required. The results of this would then produce evidence on the strength of the correlation between patients and pre-clinical models, commenting on the translational integrity of this approach. Ideally, this approach would be repeated for testing the validity in multiple cognitive domains, such as motor function, memory and spatial awareness. This strategy has the potential to provide a more robust comparison between pre-clinical models and patients with equivalent quantitative data opposed to current patient stroke scales assessing individual cognition.

## References

[bibr1-23982128231182506] Berends AC Luiten PG Nyakas C (2005) A review of the neuroprotective properties of the 5-HT1A receptor agonist repinotan HCl (BAYx3702) in ischemic stroke. CNS Drug Reviews 11(4): 379–402. 16614737 10.1111/j.1527-3458.2005.tb00055.x PMC6741728

[bibr2-23982128231182506] Berge E Whiteley W Audebert H , et al. (2021) European Stroke Organisation (ESO) guidelines on intravenous thrombolysis for acute ischaemic stroke. European Stroke Journal 6(1): I–LXII. 10.1177/2396987321989865 PMC7995316 33817340

[bibr3-23982128231182506] Bhagavati S (2015) Intravenous thrombolysis for ischaemic strokes: A call for reappraisal. Brain 138(4): e341–e341. 25281867 10.1093/brain/awu282

[bibr4-23982128231182506] Brown SGA Macdonald SPJ Hankey GJ (2013) Do risks outweigh benefits in thrombolysis for stroke? BMJ 347(7923): f5215. 10.1136/bmj.f5215 23990634

[bibr5-23982128231182506] BTS Bioengineering (2019) Nirvana – virtual reality applied to neuromotor rehabilitation. Available at: https://www.btsbioengineering.com/nirvana/ (accessed 11 August 2021).

[bibr6-23982128231182506] Butler D (2008) Translational research: Crossing the valley of death. Nature 453(7197): 840–842. 18548043 10.1038/453840a

[bibr7-23982128231182506] Chen X Wang K (2016) The fate of medications evaluated for ischemic stroke pharmacotherapy over the period 1995–2015. Acta Pharmaceutica Sinica B 6(6): 522–530. 27818918 10.1016/j.apsb.2016.06.013 PMC5071630

[bibr8-23982128231182506] Choi YH Paik NJ (2018) Mobile game-based virtual reality program for upper extremity stroke rehabilitation. Journal of Visualized Experiments 133: 56241. 10.3791/56241 PMC5931529 29578520

[bibr9-23982128231182506] Cipresso P Giglioli IAC Raya MA , et al. (2018) The past, present, and future of virtual and augmented reality research: A network and cluster analysis of the literature. Frontiers in Psychology 9: 2086. 30459681 10.3389/fpsyg.2018.02086 PMC6232426

[bibr10-23982128231182506] De Luca R Russo M Naro A , et al. (2018) Effects of virtual reality-based training with BTs-Nirvana on functional recovery in stroke patients: Preliminary considerations. The International Journal of Neuroscience 128(9): 791–796. 29148855 10.1080/00207454.2017.1403915

[bibr11-23982128231182506] Ehrenreich H Weissenborn K Prange H , et al. (2009) Recombinant human erythropoietin in the treatment of acute ischemic stroke. Stroke 40(12): e647–656. 19834012 10.1161/STROKEAHA.109.564872

[bibr12-23982128231182506] Faria AL Andrade A Soares L , et al. (2016) Benefits of virtual reality based cognitive rehabilitation through simulated activities of daily living: A randomized controlled trial with stroke patients. Journal of Neuroengineering and Rehabilitation 13(1): 96. 27806718 10.1186/s12984-016-0204-z PMC5094135

[bibr13-23982128231182506] Fisher M Feuerstein G Howells DW , et al. (2009) Update of the stroke therapy academic industry roundtable preclinical recommendations. Stroke 40(6): 2244–2250. 19246690 10.1161/STROKEAHA.108.541128 PMC2888275

[bibr14-23982128231182506] GBD 2019 Diseases and Injuries Collaborators (2020) Global burden of 369 diseases and injuries in 204 countries and territories, 1990–2019: A systematic analysis for the global burden of disease study 2019. The Lancet 396(10258): 1204–1222. 10.1016/S0140-6736(20)30925-9 PMC7567026 33069326

[bibr15-23982128231182506] Geraets CNW Veling W Witlox M , et al. (2019) Virtual reality-based cognitive behavioural therapy for patients with generalized social anxiety disorder: A pilot study. Behavioural and Cognitive Psychotherapy 47(6): 745–750. 30915939 10.1017/S1352465819000225

[bibr16-23982128231182506] Gutiérrez Zúñiga R AlonsodeLeciñana M Díez A , et al. (2021) A new software for quantifying motor deficit after stroke: A case–control feasibility pilot study. Frontiers in Neurology 12(58): 603619. 33679576 10.3389/fneur.2021.603619 PMC7928282

[bibr17-23982128231182506] Ioannou A Papastavrou E Avraamides MN , et al. (2020) Virtual reality and symptoms management of anxiety, depression, fatigue and pain: A systematic review. SAGE Open Nursing 6(4): 2377960820936163. 33415290 10.1177/2377960820936163 PMC7774450

[bibr18-23982128231182506] Isenstein EL Waz T LoPrete A , et al. (2022) Rapid assessment of hand reaching using virtual reality and application in cerebellar stroke. PLoS One 17(9): e0275220. 36174027 10.1371/journal.pone.0275220 PMC9522266

[bibr19-23982128231182506] Kelly-Hayes M Beiser A Kase CS , et al. (2003) The influence of gender and age on disability following ischemic stroke: The Framingham study. Journal of Stroke and Cerebrovascular Diseases: the Official Journal of National Stroke Association 12(3): 119–126. 17903915 10.1016/S1052-3057(03)00042-9

[bibr20-23982128231182506] Kumar A Aakriti and Gupta V (2016) A review on animal models of stroke: An update. Brain Research Bulletin 122: 35–44. 26902651 10.1016/j.brainresbull.2016.02.016

[bibr21-23982128231182506] Laver KE Lange B George S , et al. (2017) Virtual reality for stroke rehabilitation. Cochrane Database Systematic Reviews 11(11): CD008349. 10.1002/14651858.CD008349.pub4 PMC6485957 29156493

[bibr22-23982128231182506] Lee J-M Rosand J Cruchaga C (2021) A failure of forward translation? The case of neuroprotection. Vessel Plus 5: 8.

[bibr23-23982128231182506] Livingstone SA Skelton RW (2007) Virtual environment navigation tasks and the assessment of cognitive deficits in individuals with brain injury. Behavioural Brain Research 185(1): 21–31. 17727970 10.1016/j.bbr.2007.07.015

[bibr24-23982128231182506] Lourbopoulos A Mourouzis I Xinaris C , et al. (2021) Translational block in stroke: A constructive and ‘out-of-the-box’ reappraisal. Frontiers in Neuroscience 15(489): 652403. 34054413 10.3389/fnins.2021.652403 PMC8160233

[bibr25-23982128231182506] MacLellan CL Paquette R Colbourne F (2012) A critical appraisal of experimental intracerebral hemorrhage research. Journal of Cerebral Blood Flow and Metabolism: Official Journal of the International Society of Cerebral Blood Flow and Metabolism 32(4): 612–627. 22293989 10.1038/jcbfm.2012.8 PMC3318157

[bibr26-23982128231182506] Macrae IM (2011) Preclinical stroke research–advantages and disadvantages of the most common rodent models of focal ischaemia. British Journal of Pharmacology 164(4): 1062–1078. 21457227 10.1111/j.1476-5381.2011.01398.x PMC3229752

[bibr27-23982128231182506] MazeEngineers (2018) Virtual morris water maze. Available at: https://conductscience.com/maze/portfolio/virtual-reality-morris-water-maze/ (accessed 11 August 2021).

[bibr28-23982128231182506] Mills KT Stefanescu A He J (2020) The global epidemiology of hypertension. Nature Reviews Nephrology 16(4): 223–237. 32024986 10.1038/s41581-019-0244-2 PMC7998524

[bibr29-23982128231182506] Minnerup J Heidrich J Rogalewski A , et al. (2009) The efficacy of erythropoietin and its analogues in animal stroke models: A meta-analysis. Stroke 40(9): 3113–3120. 19542052 10.1161/STROKEAHA.109.555789

[bibr30-23982128231182506] Muir KW Lees KR (1995) Clinical experience with excitatory amino acid antagonist drugs. Stroke 26(3): 503–513. 7886734 10.1161/01.str.26.3.503

[bibr31-23982128231182506] National Institute for Health and Care Excellence (NICE) (2013) Stroke rehabilitation in adults NICE guideline [CG162]. Available at: https://www.nice.org.uk/guidance/cg162 (accessed 11 August 2021). 31869043

[bibr32-23982128231182506] National Institute for Health and Care Excellence (NICE) (2019) Stroke and transient ischaemic attack in over 16s: Diagnosis and initial management NICE guideline [NG128]. Available at: https://www.nice.org.uk/guidance/ng128/chapter/recommendations (accessed 11 August 2021). 31211538

[bibr33-23982128231182506] Pais-Roldán P Edlow BL Jiang Y , et al. (2019) Multimodal assessment of recovery from coma in a rat model of diffuse brainstem tegmentum injury. Neuroimage 189(21): 615–630. 30708105 10.1016/j.neuroimage.2019.01.060 PMC6642798

[bibr34-23982128231182506] Parry-Jones AR Sammut-Powell C Paroutoglou K , et al. (2019) An intracerebral hemorrhage care bundle is associated with lower case fatality. Annals of Neurology 86(4): 495–503. 31291031 10.1002/ana.25546 PMC6771716

[bibr35-23982128231182506] Rubio B Nirme J Duarte E , et al. (2013) Virtual reality based tool for motor function assessment in stroke survivors. In: Pons J Torricelli D Pajaro M (eds) Converging Clinical and Engineering Research on Neurorehabilitation, Biosystems & Biorobotics, vol.1. Berlin: Springer, pp. 1037–1041.

[bibr36-23982128231182506] Salmeron KE Maniskas ME Edwards DN , et al. (2019) Interleukin 1 alpha administration is neuroprotective and neuro-restorative following experimental ischemic stroke. Journal of Neuroinflammation 16(1): 222. 31727174 10.1186/s12974-019-1599-9 PMC6857151

[bibr37-23982128231182506] Sandercock PAG Counsell C Gubitz GJ , et al. (2008) Antiplatelet therapy for acute ischaemic stroke. The Cochrane Database of Systematic Reviews 648(3): CD000029. 10.1002/14651858.CD000029.pub2 18646056

[bibr38-23982128231182506] Schmitt YS Hoffman HG Blough DK , et al. (2011) A randomized, controlled trial of immersive virtual reality analgesia during physical therapy for pediatric burn injuries. Burns: Journal of the International Society for Burn Injuries 37(1): 61–68. 20692769 10.1016/j.burns.2010.07.007 PMC2980790

[bibr39-23982128231182506] Schneider CB Linse K Schönfeld R , et al. (2017) Spatial learning deficits in Parkinson’s disease with and without mild cognitive impairment. Parkinsonism & Related Disorders 36: 83–88. 28027851 10.1016/j.parkreldis.2016.12.020

[bibr40-23982128231182506] Selim M (2009) Deferoxamine mesylate. Stroke 40(suppl.1): S90–S91. 19064798 10.1161/STROKEAHA.108.533125

[bibr41-23982128231182506] Selim M Foster LD Moy CS , et al. (2019) Deferoxamine mesylate in patients with intracerebral haemorrhage (i-DEF): A multicentre, randomised, placebo-controlled, double-blind phase 2 trial. Lancet Neurology 18(5): 428–438. 30898550 10.1016/S1474-4422(19)30069-9 PMC6494117

[bibr42-23982128231182506] Smith CJ Hulme S Vail A , et al. (2018) SCIL-STROKE (Subcutaneous Interleukin-1 Receptor Antagonist in Ischemic Stroke): A randomized controlled phase 2 trial. Stroke 49(5): 1210–1216. 29567761 10.1161/STROKEAHA.118.020750

[bibr43-23982128231182506] Teal P Davis S Hacke W , et al. (2009) A randomized, double-blind, placebo-controlled trial to evaluate the efficacy, safety, tolerability and pharmacokinetic/pharmacodynamic effects of a targeted exposure of intravenous repinotan in patients with acute ischemic stroke: Modified randomized Ex. Stroke 40(11): 3518–3525. 19745176 10.1161/STROKEAHA.109.551382

[bibr44-23982128231182506] Thomalla G Boutitie F Ma H , et al. (2020) Intravenous alteplase for unknown time of onset stroke guided by advanced imaging: A systematic review and meta-analysis of individual patient data. The Lancet 396(10262): 1574–1584. 10.1016/S0140-6736(20)32163-2 PMC7734592 33176180

[bibr45-23982128231182506] Turner RC DiPasquale K Logsdon AF , et al. (2016) The role for infarct volume as a surrogate measure of functional outcome following ischemic stroke. Journal of Systems and Integrative Neuroscience 2(4): 210–216. 10.15761/JSIN.1000136 PMC5347398 28299202

[bibr46-23982128231182506] Withers SE Parry-Jones AR Allan SM , et al. (2020) A Multi-model pipeline for translational intracerebral haemorrhage research. Translational Stroke Research 11(6): 1229–1242. 32632777 10.1007/s12975-020-00830-z PMC7575484

[bibr47-23982128231182506] Woo D Haverbusch M Sekar P , et al. (2004) Effect of untreated hypertension on hemorrhagic stroke. Stroke 35(7): 1703–1708. 15155969 10.1161/01.STR.0000130855.70683.c8

[bibr48-23982128231182506] World Health Organisation (2020) Global health estimates 2020: Deaths by cause, age, sex, by country and by region, 2000–2019. Available at: https://www.who.int/data/gho/data/themes/mortality-and-global-health-estimates/ghe-leading-causes-of-death (accessed 11 August 2021).

